# Pre-Stimulus Activity Predicts the Winner of Top-Down vs. Bottom-Up Attentional Selection

**DOI:** 10.1371/journal.pone.0016243

**Published:** 2011-02-28

**Authors:** Ali Mazaheri, Nicholas E. DiQuattro, Jesse Bengson, Joy J. Geng

**Affiliations:** 1 Center for Mind and Brain, University of California Davis, Davis, California, United States of America; 2 Donders Institute for Brain, Cognition and Behavior, Radboud University Nijmegen, Nijmegen, The Netherlands; 3 Department of Psychology, University of California Davis, Davis, California, United States of America; Kyushu University, Japan

## Abstract

Our ability to process visual information is fundamentally limited. This leads to competition between sensory information that is relevant for top-down goals and sensory information that is perceptually salient, but task-irrelevant. The aim of the present study was to identify, from EEG recordings, pre-stimulus and pre-saccadic neural activity that could predict whether top-down or bottom-up processes would win the competition for attention on a trial-by-trial basis. We employed a visual search paradigm in which a lateralized low contrast target appeared alone, or with a low (i.e., non-salient) or high contrast (i.e., salient) distractor. Trials with a salient distractor were of primary interest due to the strong competition between top-down knowledge and bottom-up attentional capture. Our results demonstrated that 1) in the 1-sec pre-stimulus interval, frontal alpha (8–12 Hz) activity was higher on trials where the salient distractor captured attention and the first saccade (bottom-up win); and 2) there was a transient pre-saccadic increase in posterior-parietal alpha (7–8 Hz) activity on trials where the first saccade went to the target (top-down win). We propose that the high frontal alpha reflects a disengagement of attentional control whereas the transient posterior alpha time-locked to the saccade indicates sensory inhibition of the salient distractor and suppression of bottom-up oculomotor capture.

## Introduction

Our world is rich with sensory information, but our ability to process and act on it is fundamentally limited. There is a constant tension between selection of sensory information that is relevant for top-down goals versus sensory information that is perceptually salient but task-irrelevant; this has led to the frequent characterization of top-down and bottom-up attentional processes as being in competition [1]–[12]. In order to select task-relevant information, it is necessary to maintain goal-relevant attention and inhibit sensitivity to sensory stimuli that would otherwise capture attention. Conversely, being in a state that is disengaged from the current task would likely result in greater attentional capture. The central aim of the paper is to identify pre-stimulus and pre-saccadic neural activity that is predictive of whether top-down or bottom-up attentional processes win the competition for early attentional and oculomotor control.

We employed a paradigm in which attentional capture by a perceptually salient stimulus was placed in direct competition with top-down knowledge about task-relevance: the salient stimulus was never the target, but its perceptual prepotency nevertheless produced attentional and oculomotor capture on some trials [13]. This paradigm is ideal for testing the pre-stimulus and pre-saccadic brain states that give rise to greater or lesser sensitivity to bottom-up attentional capture because it directly pits top-down knowledge against bottom-up salience on a trial-by-trial basis. We used first saccades to the target (fs-target) or distractor (fs-distractor) as an index of the whether top-down or bottom-up attentional processes won the competition for selection on that trial. This method is based on evidence that shifts in covert attention precede both voluntary eye-movements and reflexive saccades (known as “oculomotor capture”) and can therefore be thought of as an overt measure of the “winner” of competition for attentional selection [14]–[18].

EEG is a non-invasive method of measuring human brain activity that provides a direct window into the variability of ongoing neural fluctuations. The oscillatory activity in the EEG is believed to reflect frequency-specific networks in the brain, while the event-related changes in the EEG reflect the reorganization of these networks in relation to event-specific computational demands [19]–[20]. In humans the presence of ongoing alpha oscillations in a region has often been found to be related to the functional inhibition or task disengagement of that region [21]. We were particularly interested in alpha (8–12 Hz) activity as a predictor of attentional capture because prior studies have implicated the involvement of this band in various aspects of visual processing and attention [22]–[28]. We used the oscillatory activity of the EEG, along with stimulus and saccade locked event related potentials (ERPs), to characterize the neural events leading to the outcome between top-down and bottom-up competition.

## Methods

### Participants

Eleven normal young adults (8 female) with a mean age of 25 years (range 18–34) participated. All subjects were right-handed as assessed by a shortened version of the Edinburgh handedness inventory [29] and had normal or corrected-to-normal vision. All gave informed consent in accord with the local ethics clearance as approved by NIH.

### Experimental Procedure

**Figure 1:**
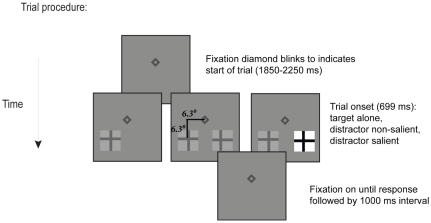
Example trial procedure. Each trial began with a blink of the fixation diamond. After a jittered interval, the visual search items appeared (illustrated here by a target in the left visual field) and subjects were free to move their eyes and indicate whether the target “t” was upright or inverted. Targets appeared alone, with a neutral distractor, or a salient distractor. Note that items are not drawn to scale for illustrative clarity.

Stimuli were composed of a ‘t’-like stimulus superimposed on a square background ( Fig. 1). Target stimuli were defined as an upright or inverted “t” and were located randomly in the left or right lower visual field (6.3° of horizontal and vertical visual angle from fixation). Targets subtended approximately .9° of visual angle at fixation. The distractor stimuli (90° rotations of target) were either low contrast (Michelson Contrast Ratio = .51; foreground luminance = 5.4 cd/m2, background luminance = 16.8 cd/m2) or high contrast (Michelson Contrast Ratio = .96; foreground luminance = .54 cd/m2, background luminance = 30.5 cd/m2); High contrast stimuli are referred to as being “salient” and low contrast stimuli as “non-salient”. The background was gray (9.8 cd/m2).

Each trial began with a fixation diamond that was on for a random interval varying between 1500–2000 ms (Figure 1). The jittered interval was used to reduce expectations regarding the onset of the visual search display and anticipatory saccades. The blank fixation screen was followed by the visual search display, which consisted of a (non-salient) target appearing either alone or with a non-salient or salient distractor. The display was visible for 600 ms which forced participants to respond rapidly and invoked more “reflexive” responses that may be more prone to error (i.e., the speed-accuracy tradeoff) [30]–[31].

Participants were instructed to fixate on the diamond until the visual search display appeared. The task was to determine whether an upright or inverted ‘t’ stimulus was present on each trial; subjects were instructed to press the left mouse key with their right index finger to indicate an “upright” choice and the right mouse key to indicate an ‘inverted” choice. There were 6 experimental conditions given by crossing 2 target locations (left, right) by 3 stimulus conditions (target-alone, neutral (i.e., target+non-salient distractor), and distractor-salient (i.e., target+salient distractor)). The fixation diamond remained visible after the stimuli offset and the next trial only began after the participant responded and 1000 ms had elapsed. The main experiment was preceded by 20 practice trials. One subject experienced a total of 384 trials, two subjects 456 trials each, and the remaining subjects experienced 504 trials. Trials were evenly divided between the 6 experimental conditions for all subjects. Compliance with the instructions was monitored by experimenter observation and an automatic pre-stimulus fixation checker. Eye position was collected using an EyeLink 2 K desk mounted system (SR Research, ON) sampling at 250 Hz. Trials were removed from subsequent analysis if fixation was not appropriately maintained and if the first saccade was not directed to one of the two possible stimulus locations. Of this subset, only correct response trials were included. This resulted in an average of 70 trials in each of the of 6 conditions per subject.

### Data acquisition and analysis

#### EEG recording

EEG was recorded from thirty two scalp electrodes located at the sites of the International 10–20 system of electrode placement. The signals were acquired using a bandpass of DC-100 Hz, and an analog-to-digital sampling rate of 1000 samples per second. The left mastoid served as the reference electrode site. The data were later referenced to a link-mastoid montage off-line.

#### EEG preprocessing

Data analysis was completed using the Fieldtrip software package (http://www.ru.nl/fcdonders/fieldtrip/), a Matlab-based toolbox for the analysis of electrophysiological data. Artifacts (e.g., trials containing premature eye movements, blinks, muscle potentials, and amplifier or electrode noise) were removed from the EEG using a semiautomatic routine. Independent component analysis [32] was used to remove any heart artifacts and eye movements not rejected by the semiautomatic routines [33]. There were no significant differences between conditions in terms of the number of trials removed. All EEG epochs were baseline corrected to a 1 sec period prior to the stimulus onset. In order to equate the number of trials per condition entered into the EEG analyses for each subject, the number of trials from each condition were matched to the condition with the fewest trials. The subset of trials included for analysis were randomly selected from all trials within a condition. This resulted in an average of 42 trials for each condition per person.

#### Time-frequency representations of oscillatory power

Time-frequency representations (TFRs) of power were calculated for each trial using a taper approach applied to short sliding time windows [34]. The data in each time window were multiplied with a Hanning taper having the length of the time window for the frequencies 2–30 Hz. A similar approach was used in [22], [35] and [27]. Our selection of frequency bands were based on the main frequency bands used to classify the spontaneous EEG [36], and prior literature [22], [27], [37]–[40].

#### ERP analysis

The stimulus locked ERP data were averaged with the sweep beginning 0.5 s before the stimuli and lasting until 0.5 s after stimulus onset. The maximum amplitudes and peak latencies of the visual and N1 components were measured. The saccade locked ERPs were averaged with the sweep beginning 0.5 s prior to the onset of the saccade until 0.5 s after. Both the stimulus and saccade-locked ERPs were baseline corrected using the mean time 1 sec prior to stimulus onset.

## Results

We were specifically interested in trials where top-down knowledge was pitted against bottom-up attentional capture. The analyses of primary interest therefore involved trials in which a salient distractor was present and the first saccade was either directed to the target, or captured by the salient distractor. Analyses of neutral (i.e., non-salient distractor) trials were also included to control for any effects related to making a first saccade to the target or distractor, irrespective of distractor salience (i,e., the random selection of either stimulus). These analyses were important for interpreting differences between trials with first saccades to the target versus distractor in the distractor-salient condition.

### Behavioral data: first saccade

All data in each of the three trial types of interest (i.e. target-alone, neutal, and distractor-salient) were first divided based on whether the first saccade on a given trial was directed to the target (fs-target) or the distractor (fs-distrctor). A .5 proportion of fs-target trials would indicate that the subject randomly selected the target or the distractor on each trial. Values greater than .5 indicate a bias to saccade to the target first and values less than .5 indicate a bias to saccade to the distractor first.

As expected, all subjects had 100% fs-target trials in the target-alone condition when there was no distractor competition. Similarly, all subjects had a proportion near .5 (ranging from .46–.53) in the neutral condition when targets and distractors were matched in perceptual salience. Interestingly, the proportion of fs-target trials was more heterogeneous when the distractor was salient: for most subjects (7/11), there was a bias to saccade to the distractor first (i.e. .13–.45 fs-target trials), but for 4 subjects, the bias was to saccade to the target first (i.e. .52–.86). This heterogenity in strategy suggests that some subjects were more susceptible to bottom-up attentional capture by the salient distractor and others were able to exert greater top-down control. Nevertheless, despite differences in the proportion of fs-target and fs-distractor trials when the distractor was salient, fs-target trials resulted in shorter RTs, (*fs-target* = 1131.9 ms; *fs-distractor* = 1266.9 ms; F(1,10) = 29.2, p<.0005). This pattern was seen in all subjects (fs-target minus fs-distractor ranged from −48 ms to −587 ms; p<.005 with a binomial test); the difference in accuracy was not significant, (*fs-target* = 93.9% ms; *fs-distractor* = 89.9% ms; F(1,10) = 2.1, p≤.17). The fact that fs-target trials resulted in faster RTs was unsurprising since fs-distractor trials required a second saccade to fixate the target. Nevertheless, the result demonstrates that the failure to make a first saccade to the target, despite prior knowledge that salience was never a property of the target, resulted in a cost in performance.

**Figure 2:**
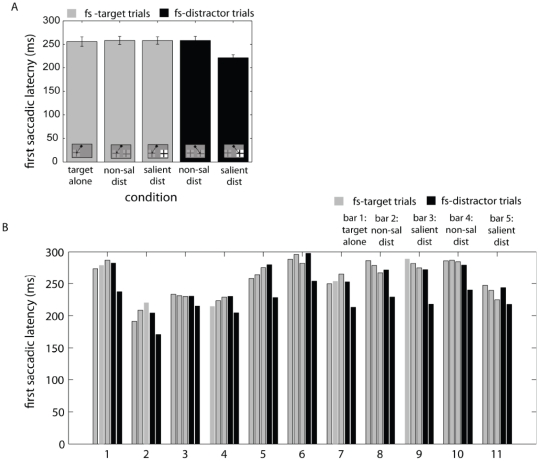
The latency of saccades. First saccade latencies in each experimental condition in A) group and B) individuals. Error bars on group data are standard error of the mean. First saccade latencies were significantly faster for fs-distractor trials in the distractor-salient condition. This suggests that the salient distractor produced automatic oculomotor capture.

Fs-target trials were more efficient and this suggests that fs-distractor trials were due to a failure of top-down attentional control to direct the first saccade away from the salient item, which was known to be a non-target. Consistent with the notion that fs-distractor trials were due to involuntary oculomotor capture, saccades to the salient distractor had shorter latencies than any other trial type, including target-alone trials (all t(10)>10, p<.0001; Figure 2AB). This pattern was seen in all individuals (Figure 2B). Thus, despite individual differences in the likelihood of executing a saccade to the target versus the salient distractor first, fs-distractor trials represented instances where automatic bottom-up selection of the salient item won the competition for attention despite prior knowledge of its task-irrelevance. These results are similar to previous findings where behavioral and oculomotor responses from a similar paradigm are more fully explicated [13].

### EEG data

Behavior on distractor-salient trials directly measured the outcome of competition between top-down knowledge and bottom-up attentional capture; the behavioral data demonstrated trial-by-trial differences in whether oculomotor control of the first saccade was won by top-down (i.e., fs-target trials) or bottom-up (i.e., fs-distractor trials) attentional processes. Next, we used EEG data to determine the brain states that led to oculomotor capture by the salient distractor, despite knowledge that it was never the target. Importantly, to rule out other variables that might be related to fs-distractor eye-movements irrespective of the salient distractor, we directly compared the ERP and TFR results in the distractor-salient and neutral conditions using a 2×2 repeated measures ANOVA with factors of first saccade (to distractor or target) and salience (distractor salient or non-salient). We restricted our analysis to the electrode site, time interval and frequency band that showed the greatest amount of activity based on the grand-average of the data across all the conditions. We then looked within that electrode site and interval for condition-specific effects.

### Stimulus locked analysis

#### Pre-stimulus determinants

alpha activity is indicative of a bottom-up win.

**Figure 3:**
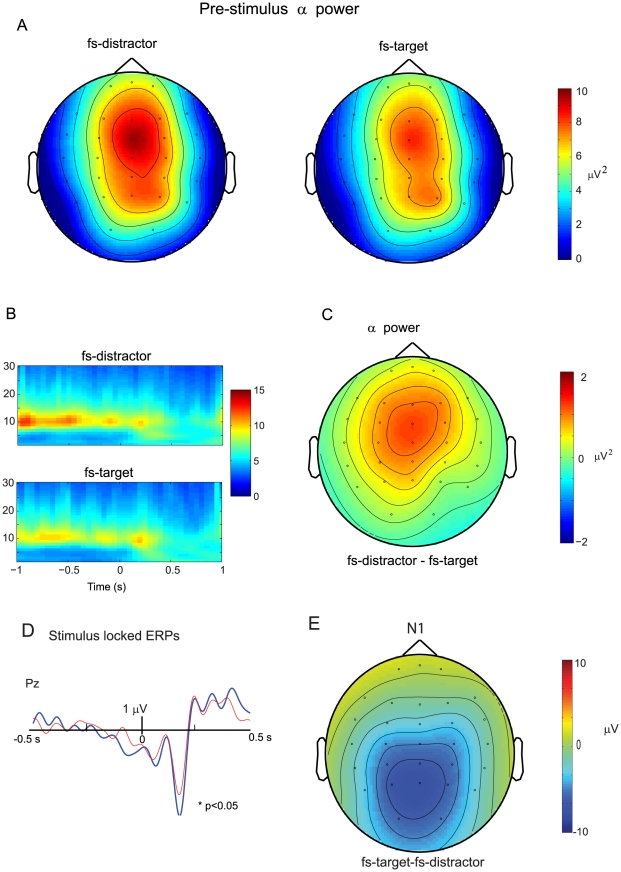
Pre-stimulus (−1 -to 0 s) alpha activity is indicative of a bottom-up win. A) Grand Average of the topography of pre-stimulus alpha power (8–12 Hz) for fs-distractor trials (left) and fs-target trials (right). The alpha activity is maximal at the central frontal electrodes. B) The time-frequency representations of fs-distractor (top) and fs-target trials (bottom) at the frontal central FCz electrode. C) The topography of the difference in pre-stimulus alpha- power between fs-distractor and fs-target trials. There was significantly greater pre-stimulus alpha in fs-distractor than fs-target trials. D) The stimulus locked N1 response. The peak amplitude of visual N1 response occurring at 0.175 s was bigger for fs-target trials (blue line) than fs-distractor trials (red line). E) The topography of the N1 response.

Pre-stimulus power spectra was compared for fs-target and fs-distractor trials to determine the oscillatory activity leading to oculomotor capture by the salient distractor. The topography of pre-stimulus (−1 to 0 sec) alpha activity (8–12 Hz) for both fs-target and fs-distractor trials had a fronto-central distribution with a maximal value at the ‘FCZ’ electrode (Figure 3A). Our statistical analysis revealed a significant interaction between the first saccade and distractor salience, (F(1,10) = 5.853, p = .036,): In the one second interval prior to the onset of the search array when the distractor was salient, alpha activity was greater for fs-distractor than fs-target trials, (8.68 µV^2^ vs 10.1 µV^2^, see figure 3B and C). In contrast, there were no differences in pre-stimulus alpha power between fs-target and fs-distractor trials when the distractor was *not* salient (9.43 µV^2^ vs.9.49 µV^2^). These results suggest that pre-stimulus frontal alpha corresponded to a disengagement from task goals that resulted in greater sensitivity to sensory events. When the distractor was salient, this led to oculomotor capture by the salient distractor, despite knowledge that it was a non-target. Importantly, when the distractor was non-salient, sensory information for the two objects was equal and pre-stimulus alpha did not predict the destination of the first saccade.

### ERP analysis

The stimulus locked ERP wave forms can be seen in Fig. 3D. The peak amplitude of the visual N1 response occurred at 175 ms. This peak amplitude had a maximal topography over the posterior Pz electrode (Fig. 3E). Statistical analysis on the mean amplitude (from 80 to 200 ms) of the N1 revealed a significant interaction between the first saccade and stimulus salience, (F(1,10) = 7.079, p = .024,): the mean peak of the N1 was bigger in fs-target (−6.16 µV) than fs-distractor trials ( −5.29 µV), when the distractor salient, but the mean amplitude of N1 response in fs-target (−5.20 µV) and fs-distractor (−5.52 µV) trials was not significant when the distractor was not salient.

The N1 occurred before the saccade was executed and its attenuation in fs-distractor trials could reflect the absence of rapid top-down selection of the target stimulus. Numerous studies have demonstrated that sensory-specific N1 components at posterior electrodes are enhanced in response to visual stimuli at attended versus unattended locations (e.g. [41], [42]).

### Pre-saccadic determinants of top-down control

**Figure 4:**
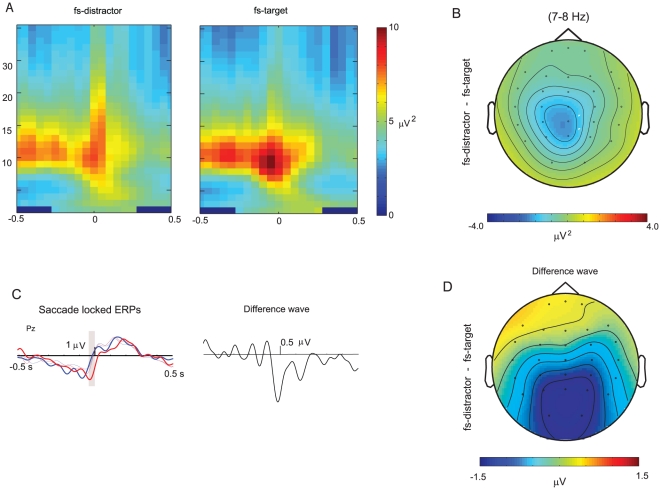
Transient increase in theta/alpha (7–8 Hz) activity just prior to top-down saccade. A) There was a transient alpha increase locked to the saccade onset. This transient increase was significantly larger for fs-target trials. B) The topography difference of the transient theta/alpha increase (mean −0.1 to 0 s) between fs-distractor and fs-target trials. C) The saccade locked ERPs for fs-distractor (red) and fs-target (blue) trials in both salient (thick lines) and none salient distractor (thin lines) conditions. A slow negative drift preceded the onset of all the saccades. The difference wave between fs-target and fs-distractor trials revealed a negative deflection. D) The topography of the negative deflection observed the in fs-target- fs-distractor difference wave.

In addition to the pre-stimulus alpha, we were also interested in whether oscillatory activity differed between fs-target and fs-distractor trials prior to the initiation of the saccade. In order to avoid confounds associated with differences in saccade latency between conditions (see behavioral results above), we examined brain activity aligned to the onset of the saccade. Here, there was a transient increase in activity in the high theta/alpha range (7–8 Hz) with maximal distribution over the parietal electrode ‘Pz’ starting 100 ms prior the onset of the saccade (figure 4A and B). The statistical analysis of this transient pre-saccadic burst revealed an interaction between the first saccade and salience, (F(1,10) = 7.544, p = .021): the mean saccade locked alpha burst (−0.1 to 0 S) was significantly larger for fs-target than fs-distractor trials,when the distractor was salient (8.18 µV^2^ vs 6.1 µV^2^ ) but not when the distractor was non-salient ( 6.75 µV^2^ vs 7,29 µV^2^ ). We conjecture that this transient increase in alpha activity plays a role in top-down oculomotor control that prevented a reflexive saccade to the more salient distractor.

In addition to the oscillatory analyses, we also examined the saccade locked ERPs for fs-distractor and fs-target trials (Figure 4 C left panel). The results show a slow negative drift building up to a positive deflection just prior to the onset of a saccade. Statistical analysis of this negative drift (mean amplitude from −0.5 to −0.1 S) found a significant interaction between the first saccade and salience, (F(1,10) = 4.962, p = .050, η2 = .332): fs-target trials had a larger negativity than fs-distractor trials when the distractor was salient, (−0.86 µV vs −0.50 µV ), but not when the distractor was non-salient, (−0.65 µV vs −0.67 µV ). The difference wave between fs-targets and fs-distractor trials revealed a negative deflection (Figure 4 C right panel) with a maximal topography over the posterior parietal regions. The ‘Pz’ electrode was the site of maximal statistical difference between fs-target and fs-distractor conditions, but the topography was clearly more posterior (Fig. 4 D) than that of the pre-saccade alpha (Fig. 4B); the ERP effects were similar in ‘O1’ and ‘O2’ (not shown). Statistical analysis of this deflection (mean amplitude from −0.05 to 0.05 S) also revealed a significant interaction of the first saccade and salience: (F(1,10) = 7.82, p = .019 ):The fs-distractor trials during salient distractors had a negative inflection locked to the onset of the saccade (−1.04 µV ), but the ERP deflection was positive for all the other saccades ( mean 0.35 µV ).

The pre-saccadic parietal alpha and posterior ERP waveform together leads us to suggest that top-down control is implemented in multiple mechanisms that determined whether the first saccade would be directed to the less salient target, or be captured by the salient distractor.

## Discussion

To directly pit top-down knowledge against bottom-up salience, we employed a task where a non-salient visual target sometimes appeared with a salient distractor. The salient distractor produced oculomotor capture on some trials despite never being the target. This paradigm allowed us to investigate the trial-by-trial neural activity that predicted the outcome of bottom-up and top-down attentional competition. Our results demonstrate several distinct processes related to top-down and bottom-up selection and suggest that multiple mechanisms control top-down attention on a trial-by-trial basis.

We found that an increase in pre-stimulus alpha activity over frontal-central regions was predictive of subsequent attentional capture by a salient distractor. Previous studies have found an alpha increase in a particular region to be indicative of the functional inhibition/disengagement of that region [22], [23], [24]–[27], [43].

Our current results are consistent with frontal alpha indicating task-disengagement as trials with greater frontal alpha resulted in oculomotor capture by the salient distractor. The frontal-central topography of the pre-stimulus alpha activity on fs-distractor trials could reflect the disengagement of the frontal-eye fields (FEF). FEF is involved in top-down voluntary control of saccades and attention [44]–[52]. Greater pre-stimulus alpha in FEF could indicate its disengagement from the task that would then increase the likelihood of attentional and oculomotor capture by a task-irrelevant salient stimulus.

We also reported an attenuated N1 response to the stimulus-array for fs-distractor trials relative to trials when top-down processes won the competition for the first saccade. Numerous studies have demonstrated that sensory-specific N1 components at posterior electrodes are enhanced in response to visual stimuli at attended versus unattended locations [53], [55]. The attentional modulations of early visual ERP components are thought to reflect location-specific sensory gating mechanisms that bias visual processing in favor of stimuli at the current focus of spatial attention. In particular, the N1 is involved in processes of target discrimination [54]. The bigger N1 on fs-target trials therefore suggests that greater task-relevant processing locked to the appearance of the stimulus occurred on trials where the target was fixated first.

In addition to the stimulus-locked effects there were also two saccade-locked results that differentiated between trials where the first saccade was directed to the target compared to the distractor. First, there was a central-parietal alpha burst just preceding the onset of the first saccade that was greater in amplitude for saccades to the target. This saccade-locked alpha was specific to distractor-salient trials and could index the transient inhibition of the prepotent response to saccade to the more salient distractor; when inhibition was successful, top-down processes won the competition for selection and the first saccade was directed to the target. The intraparietal sulcus contains an attentional priority map and is involved in saccadic control [5], [8], [51], [53]–[54], [56]–[59] and is a good candidate for being the source of the inhibitory control signal seen here.

Second, the difference of saccade-locked ERPs between the trials of fs-targets and fs-distractors revealed a negative component locked to the onset of fs-distractors during salient distractors. To the best of our knowledge this is the first report of such a component. A qualitative inspection of the saccade locked ERPs suggests that this negative deflection is due to a latency shift in the slow negative drift building up to a potential pre-ceding the saccade to a salient distractor. More work needs to be done to reveal what the functional significance of this latency shift is in the context of top-down vs. bottom-up saccade initiation.

### Conclusion

We describe several neural processes related to the outcome of bottom-up vs. top-down selection processes as indexed by the first oculomotor response in a visual search task. We report both stimulus- and saccade-locked processes in scalp EEG and ERP that differentiate between trials in which the first saccade is captured by a salient, but task-irrelevant stimulus, versus voluntarily directed to the target. Some of these processes exert their influence on the outcome well before the onset of the stimulus whereas others occur after the stimulus array appears, but before the saccade is executed. Given the time course and scalp topography of these processes we conjecture that they reflect the activity of distinct neural networks.
